# A Preliminary Real-Time and Realistic Simulation Environment for Percutaneous Coronary Intervention

**DOI:** 10.1155/2015/183157

**Published:** 2015-03-23

**Authors:** Jianhuang Wu, Haoyu Wang, Peng Zhang, Xin Ma, Qingmao Hu

**Affiliations:** Shenzhen Institutes of Advanced Technology, Chinese Academy of Sciences, Shenzhen 518055, China

## Abstract

Percutaneous coronary intervention (PCI) is a minimally invasive surgery procedure that is widely used in the treatment of coronary artery disease. This procedure requires interventional cardiologists to have high proficiency and therefore demands an extensive training period in order to ensure successful surgical outcome. In this paper, a realistic and real-time interactive simulator for training PCI procedure is presented. A set of new approaches for core simulation components is devised and integrated into the simulator. Trainees can interact with the virtual simulation environment with real instruments and essential maneuvers encountered in real PCI procedure. Although presently targeted at PCI, our simulator could be easily extended to mimic the necessities of any vascular interventional radiology procedures by updating vascular anatomy. Preliminary validation of the proposed physical model of instruments is conducted on vascular phantom to demonstrate its performance and effectiveness.

## 1. Introduction

Coronary artery disease (CAD) is the most common type of heart disease and is one of the leading causes of death worldwide. This disease is caused by plaque buildup inside the walls of the coronary arteries, which narrows or blocks the blood vessels and reduces the flow of oxygen-rich blood to the heart. Percutaneous coronary intervention (PCI), which is also known as coronary angioplasty, is a minimally invasive surgery procedure widely used to open the narrowed or blocked arteries to improve or restore blood flow to the heart muscle.

During PCI, interventional cardiologists percutaneously insert long and thin flexible devices (such as guide wire and catheter) into patient's blood vessel from a tiny incision often made in the femoral artery or radial artery and manipulate the devices through blood vessel network under the guidance of X-ray imaging from outside the body. Once reaching the sites of coronary artery narrowing or blockage, a balloon deployed at the leading tip of the catheter is inflated to compress the plaque against the artery wall. Usually, a stent (small wire mesh tube) is placed at the area of blockage, acting as a scaffold to keep the artery open permanently.

Performing a PCI procedure is very difficult due to several factors. (1) The vessel network is of high complexity with a great variety of sizes and anatomical configurations, and the characteristics of vascular lesions vary significantly between individuals. (2) The devices such as guide wire are inherent high flexibility (e.g., low resistance to bending). (3) The devices inside the vasculatures are invisible to cardiologists' eyes and the vessels are seen on the X-ray images only when contrast dye is injected to the patient's vessels; most important, due to the morality and safety reasons (overexposure to X-rays can result in leukemia and cancer), X-ray imaging is only performed when really necessary, for example, at some critical treatment phases or positions such as bifurcations, to help make decisions for subsequent device manipulations or to confirm the diseased location and situation. As a result, most of the time, the cardiologists are blind to moving the devices while heavily relying on their sense of touch feeling of the devices at one proximal end in their hands. (4) The procedure involves some risks (e.g., vessel rupture) and complications can occur even when the most experienced and skilled cardiologist performs the procedure.

Considering the above issues, interventional cardiologists need a great deal of training to acquire necessary skills such as good hand-eye coordination of instruments, guide wires and catheters, and accurate interpretation of complex three-dimensional (3D) anatomy and pathological conditions from two-dimensional (2D) images to ensure safe and health outcomes. Traditionally, this medical training is obtained through a one-on-one interaction between the skilled cardiologist mentor and the trainee using live patients as teaching materials. However, such “see one, do one, teach one” apprenticeship training model is a high-cost, unstructured, time-consuming, and resource-intensive process with no objective assessment of proficiency [1, 2]. Most importantly, learning on patients is risky and unethical for the patients and is no longer acceptable in the world today. Other traditional training methods either on animals and cadavers or on phantoms with real devices and X-ray equipment also suffer from ethical problem, cost, and radiation exposure as well as the constraint of reusability.

Interactive computer-based virtual environment provides promising solutions to overcome many limitations of the traditional training models. In a virtual environment of replicating patient anatomy and the effects of virtual devices and by interacting with sensory inputs or real medical devices, trainees can understand the 3D relationships among anatomical structures and develop other required knowledge and skills at anytime and anywhere without exposing themselves to invasive environment. In addition, trainees can learn to manage intraoperative accidents such as artery rupture with making mistakes without putting patients at risk. Beyond the training purpose, experienced cardiologist can rehearse the PCI procedure on patient-specific image data to evaluate technical feasibility and to foresee any potential challenges that may occur in actual surgical operation. Furthermore, virtual simulator could also serve as an important component of objective certification of skills and continuing medical education to learn novel surgical techniques in a risk-free environment.

However, developing a high-fidelity and real-time immersive virtual environment is a particularly challenging task. Several companies and research groups devoted many efforts to virtual vascular interventional radiology simulators in the past years [3–9]. NeuroCath is a simulation environment for interventional neuroradiology procedures, aiming at pretreatment planning of interventions and training of interventional neuroradiologists. This system used geometric primitives like cylinder to approximately represent vessel surface, leading to discontinuity in vascular branches with unrealistic visual quality, and used linear finite element method (FEM) to model catheter. ICTS [4] is an interventional cardiology training system which is intended not only for learning but also for introducing new devices to cardiologists. ICTS applied NURBS to geometrical modeling of arteries and developed a multibody model to physical modeling of catheter. The catheter instruction system (CathI) is another computer-based training system to simulate both the patient and the catheter laboratory [5]. Mentice VIST [6] and Simbionix ANGIO Mentor [7] are two commercial endovascular simulators to provide training solutions to trainees of all levels and across multiple disciplines including interventional cardiology and interventional neuroradiology.

In addition to the above-mentioned systems, a good deal of efforts have been put towards modeling of instruments especially guide wire [10–19]. Alderliesten et al. [10, 11] modeled the guidewire as a set of connected rigid rods with several energies including friction. This model requires iteratively computing the minimum of the global energy and consequently has a low efficiency, resulting in being unsuitable for real-time simulation. Lenoir et al. [12] devised a composite model based on incremental FEM model [13] to simulate the interactions between guide wire and catheter. Luboz et al. [14] modeled the guide wire as a hybrid mass-spring particle system and introduced extra bending forces to make the guide wire stay inside the vasculature. Recently, Cosserat theory is applied to model guide wire [16, 18]. The theory is proposed for continuum mechanical modeling and takes into account all possible deformations of one-dimensional bendable object.

In this paper, a computer-based real-time and realistic simulation environment for PCI training is presented. It consists of a high-quality surface modeling and a robust centerline extraction for vascular structures. In addition, a realistic physic-based elastic rod model of virtual instruments is proposed. The rod model is discretized into different resolutions that are dynamically adaptive to the diameter and curvature of the vessel and thus can behave well even under highly complex vascular circumstances. At the collision response stage, a force correction strategy is proposed to dynamically adjust the elastic force between the guide wire or catheter and the vessel wall, being able to avoid frequent collision detections to save a great deal of time. Additionally, various rendering techniques based on modern graphics processing units (GPUs) are developed in our virtual system to achieve a real-time and realistic visualization including X-ray effect. Most importantly, our system is designed in such a way that real PCI instruments including guide wires and catheters can be used to simulate the requirements of the catheterization laboratory. Based on the above components, the presented simulation environment allows core skills training of PCI such as hand-eye coordination of catheter/guide wire insertion and navigation by pulling, pushing and twisting, contrast dye injection, C-arm manipulations, and so on.

The rest of this paper is organized as follows: details of our simulator are described in Section 2, followed by experimental results and discussion in Section 3. Finally, conclusion and comments on future works are given in Section 4.

## 2. Methodology

In this section, we firstly give a brief architecture overview of the presented PCI simulation system. Secondly, a high-quality geometric modeling method for vascular structures is introduced. Thirdly, a centerline extraction method for vasculatures is presented. Fourthly, a proposed physic-based instrument modeling is described. Fifthly, we introduce the graphic rendering techniques used in our simulation. Finally, short descriptions are given to the hardware part of the simulation system.

### 2.1. System Overview

**Figure 1:**
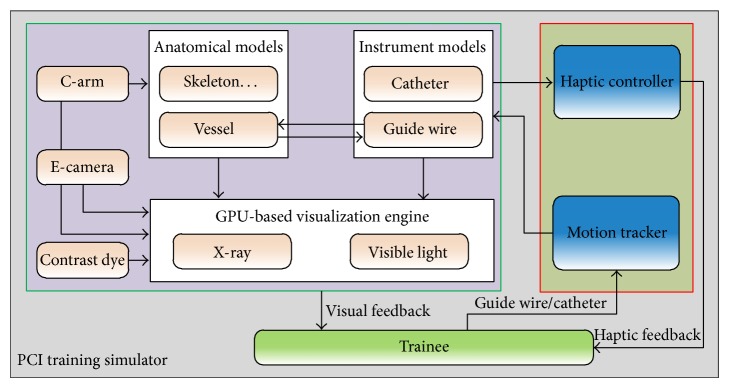
System overview of the PCI simulation environment.

The presented simulation environment not only offers trainees a realistic visual feedback similar to what an interventional cardiologist could see on the fluoroscope monitor but also provides a haptic apparatus that trainees could efficiently interact with the simulation in a natural way as done in real catheterization procedures. The architecture and the relations among different components of our simulation system and the user are shown in Figure 1. The system mainly consists of two subsystems, hardware component and software component. The former is composed of a haptic controller and a motion tracking device, while the latter comprises three modules, namely, 3D anatomical models, physical instrument models, and GPU-based visualization engine. The engine provides two rendering modes, that is, X-ray and visible light, and is responsible for rendering all the objects in the virtual environment, for example, C-arm, contrast dye, and scenes that the virtual endoscopic camera sees.

For our development, Microsoft Visual Studio 2010 is used as the main programming platform and the Microsoft Foundation Class library and Windows API are applied to build the graphical user interface (GUI) of the software component. All the algorithms in subsequent sections are implemented in C++, while the graphic programming is based on the OpenGL graphic library and GLSL shading language.

### 2.2. Geometric Modeling

Anatomical modeling is the first step toward the development of a simulation environment. A high-quality modeling makes it possible to achieve a realistic virtual scenario. In medical visualization, surface modeling of anatomical structures usually resorts to the marching cube technique [20]. However, this technique often produces a low quality surface containing strong aliasing artifacts and huge amount of triangular primitives. The artifacts not only degrade the fidelity and realism of the representation of the anatomical structures but also affect the external force computed in the physical modeling of instruments, while the quantity of triangles decreases the rendering and collision detection speed. In our previous works [21, 22], an approach of scale-adaptive surface modeling for vascular structures is proposed. This approach could not only represent the underlying data with high fidelity but also achieve a good trade-off between the visual quality and the number of triangles of the generated surface. The main stages of the approach may be described as follows (more details can be found in [21]).

**Figure 2:**
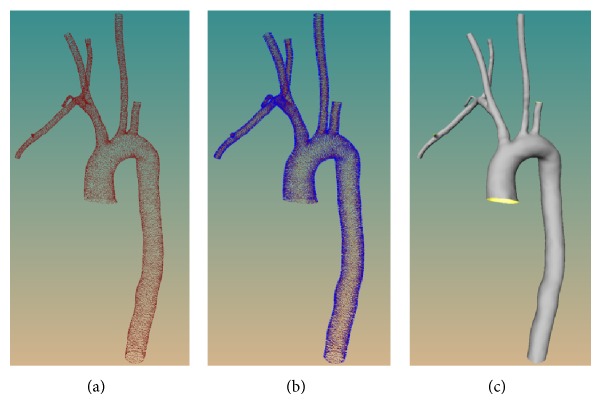
Geometric modeling of vasculature. From (a) to (c) are point set obtained by upsampling, vascular normal vector field, and resulting vascular surface, respectively.

- Point cloud extraction and adaptive upsampling: the input of this stage is a 3D volume of segmented vessel image data, and the output is a 3D point set with high densities, as shown in Figure 2(a).
- Normal vector field construction: this stage has two substages. First is calculating the normal vector of each point based on covariance analysis of the point's* k-nearest* neighboring points. Second is making all the directions of the vectors consistent via normal propagation under the help of minimum spanning tree, as illustrated in Figure 2(b).
- Indicator function construction: the function aims at implicitly describing the underlying 3D point set, which is achieved by solving a Poisson equation constructed from the normal vector field.
- Curvature-dependent polygonization: this stage also contains two parts, mesh expanding and gap stitching. In the former part, an initial mesh composed of six triangles surrounding a given point, whose edge lengths are adaptively determined according to the local curvature radius of the point, is firstly constructed and is subsequently expanded by gradually growing triangles from its boundary edges. The latter part is to fill the gaps yielded in the mesh generation. The gap is sewed by triangulating the polygon formed from the boundary edge of the gap.

### 2.3. Centerline Extraction

**Figure 3:**
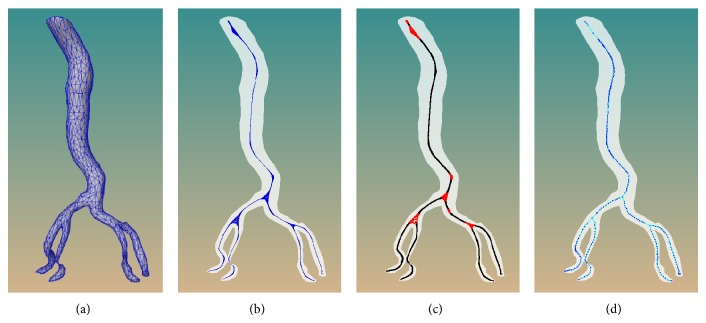
The procedure of centerline extraction method. From (a) to (d) are the input aorta vascular model, the subdivided mesh after contraction (in blue), the initial joint clustering after principal component analysis, and the output curve skeleton after fitting by cubic B-spline, respectively.

Centerline or curve skeleton, a means of compactly representing the topology of 3D objects, is useful in many medical applications. In [23], centerline is applied to simulate one-dimensional fluid flow which moves along the centerline of the vessel. In our simulation environment, the centerline is used to plan the flight path of endoscopic camera and hierarchy vessel labeling. The centerline extraction method utilized in this work is an improved version of our previous work [24]. This method is composed of three steps, as shown in Figure 3.

**Step 1 (mesh contraction and local subdivision).**

The mesh contraction procedure based on [25] is a global optimization process to shrink the input mesh to a thin mesh with unchanged topological connectivity. This procedure is operated via iteratively solving the following linear equations:

(1) $$\begin{array}{c} \left[\begin{array}{c} W_{L}L\\ W_{H} \end{array}\right]V^{\prime }=\left[\begin{array}{c} 0\\ W_{H}V \end{array}\right], \end{array}$$

where *W*_*L*_ and *W*_*H*_ are the diagonal weight matrices balancing contraction and attraction constrains; the *i*th diagonal element of *W*_*L*_(*W*_*H*_) is represented by *W*_*L*,*i*_(*W*_*H*,*i*_). *L* is the *n* × *n* Laplacian matrix with cotangent weights. The iterative contraction process works as follows, where *t* is the iterative number:

- solving *V* ^*t*+1^ through $\left[\begin{array}{c} W_{L}^{t}L^{t}\\ W_{H}^{t} \end{array}\right]V^{t+1}=\left[\begin{array}{c} 0\\ W_{H}^{t}V^{t} \end{array}\right]$,
- updating *W*_*L*_^*t*+1^ = *s*_*L*_ *W*_*L*_^*t*^ and $W_{H,i}^{t+1}=W_{H,i}^{0}\sqrt{A_{i}^{0}/A_{i}^{t}}$, where *A*_*i*_^*t*^ and *A*_*i*_^0^ are the current and original one-ring neighborhood, respectively, and *s*_*L*_ is a speed-up coefficient of the contraction matrix,
- constructing the new Laplacian matrix *L* ^*t*^ with the newly solved vertex position *V* ^*t*+1^.

The initialization of *W*_*L*_^0^, *W*_*H*_^0^, and *s*_*L*_ is set as suggested in [25]. The iterative process stops when the volume of the input model reaches nearly zero.

After obtaining the contracted mesh, we extract the skeleton on the point domain in the subsequent two steps, in contrast to the [25] on the mesh domain. Thus, before proceeding, an adaptive loop subdivision scheme [26] is performed on the obtained contracted mesh to increase the local density of point set.

**Step 2 (local principal component analysis (PCA) and joint clustering).**

The identification between the joint points and the branch points is essential for centerline extraction from vascular point set. Here, PCA is employed to assist the determination. Given a point *p*_*i*_ a function *f*(*p*_*i*_) is defined as follows:

(2) $$\begin{array}{c} f\left(p_{i}\right)=\frac{\lambda_{1}}{\lambda_{1}+\lambda_{2}+\lambda_{3}}, \end{array}$$

where *λ*_*i*_  (*i* = 1,2, 3) is the *i*th largest eigenvalue of the 3 × 3 covariance matrix for *p*_*i*_. Then, if *f*(*p*_*i*_) is smaller than a user-specified threshold *ψ*_*f*_, the point *p*_*i*_ is a joint point, and a branch point otherwise. It is noted here that the *ψ*_*f*_ should be parameterized a little larger to ensure that all joint points are identified. In our work, *ψ*_*f*_ is empirically initialized as 0.8, achieving better results for a considerable diversity of vasculatures.

The joint clustering procedure [24] is then implemented to collect points belonging to the same cluster. For an input joint point, the procedure firstly checks whether it has been clustered. If not, a new cluster is generated. Then the input joint is assigned to the cluster and flagged as clustered. Simultaneously, an empty queue is produced and the input joint point is pushed back into it. Subsequently, the head element of the queue is popped, followed by checking, assigning, and flagging again. Traversing the neighboring points of this element, if the neighbor point is a joint and has not been flagged, push it to the queue. The clustering procedure stops until the queue is empty.

**Step 3 (skeleton growing and optimization).**

Skeleton grows from seed, which is obtained by computing the joint cluster's center and searching the nearest branch point to the center in each branch. The skeleton growing will stop when there are no more points ahead in the growing direction or two growing directions meet each other. After the skeleton procedure, a complete curve skeleton is generated. Then the skeleton is optimized by performing cubic B-spline fitting.

### 2.4. Physical Modeling

**Figure 4:**
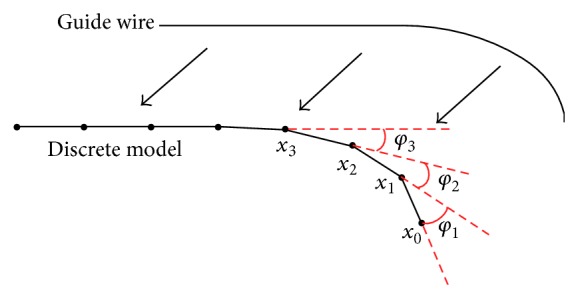
Discrete model of guide wire.

Similar to the geometric modeling of anatomical structures, physical modeling of interventional instruments is also an essential element in the development of a simulation environment with high realism. In the current development of our simulation system, the instrument models only include catheter and guide wire. These two models use the same modeling approach. Therefore, for brevity of description, hereafter in this section, we use guide wire to describe the proposed physical modeling approach. In this work, the guide wire is represented as a chain of rigid rods which tend to bend or twist rather than stretch. They are linked by adjacent joint points where material characteristics could be integrated. As indicated in Figure 4, with proper setting of intrinsic bias angle *φ*_*i*_ on every joint point, this model could represent any types of guide wires with different intrinsic shapes and deforming configurations.

To achieve a more robust and realistic simulation in vascular circumstance with high geometrical complexity, we propose an adaptive discretizing algorithm to dynamically discretize or merge the rods. Let *n*_*d*_ denote the times of discretization. It is calculated as follows:

(3) $$\begin{array}{c} n_{d}=\left\lfloor \log\frac{\bar{l_{t}}}{\left(r_{c}+0.5*d_{v}\right)\sqrt{2*\left(1-\cos\varphi_{m}\right)}}\right\rfloor , \end{array}$$

where *d*_*v*_ denotes the diameter of vessel and $\bar{l_{t}}$ is the average length of the rods. *r*_*c*_ is the radius of curvature. *φ*_*m*_ is the maximum bias angle of adjacent rods. It is clearly observed that *n*_*d*_ is closely related to the diameter and curvature of the vessel. When a guide wire advances through a high-curvature or small-diameter vascular region, *n*_*d*_ would be positive and each rod splits into 2^*n*_*d*_^ smaller ones to avoid vascular penetration and visual artifacts caused by low resolution modeling. On the contrary, when the guide wire reaches a low-curvature and large-diameter area, the calculated *n*_*d*_ is negative and 1/2^*n*_*d*_^ rods merge as one to decrease the number of joint points whose positions need to be calculated afterwards. To decrease computational cost, *n*_*d*_ is calculated in preprocessing stage instead of being calculated in real-time simulation and corresponding trigger points for splitting/merging are planted in certain area for further discretization/merging.

In our approach, the updated position of the joints is calculated based on the principle of minimum potential energy which indicates that when the total potential energy of a system reaches minimum, its equilibrium state is achieved. The guidewire-vessel system's potential energy is defined as follows:

(4) $$\begin{array}{c} E_{\text{total}}=E_{g}+E_{v}. \end{array}$$

Here, *E*_total_ is the sum of two parts: the bending energy of the guidewire *E*_*g*_ and the elastic energy of the vessel wall *E*_*v*_ which are both generated by the deformation. In this work we define the bending energy as follows:

(5) $$\begin{array}{c} E_{g}=\overset{n}{\underset{i=1}{\sum }}\frac{1}{2}C_{i}{\left(\theta_{i}-\varphi_{i}\right)}^{2}, \end{array}$$

where *θ*_*i*_ denotes the angle between adjacent rods of point *x*_*i*_, while *φ*_*i*_ represents the intrinsic bias angle of *x*_*i*_, as illustrated in Figure 4.

The energy of the vessel wall *E*_*v*_ is defined as

(6) $$\begin{array}{c} E_{v}=\overset{n}{\underset{i=1}{\sum }}\frac{1}{2}k_{v}d_{i}^{2}, \end{array}$$

where *k*_*v*_ denotes the elastic coefficient of the vessel wall. *d*_*i*_ is the depth of the vessel wall's deformation by collision.

To obtain the guide wire's equilibrium position, it is needed to iteratively calculate those joint points' displacements which make *E*_total_ reach its minimum. In each iteration, the external elastic force applied to the guide wire's joint point needs to be calculated for the subsequent computation. In previous methods [11, 14], collision detection needs to be performed in each iteration to get that external force. Therefore, it is time-consuming and inefficient and is unsuitable for a real-time simulation.

In this work, we propose a force correction algorithm to accelerate the convergence of the iteration. Instead of conducting collision detection at each iteration, we apply the outcome of the previous iteration to correct the elastic force which is in return used as the input of the next iteration based on the following equation:

(7) $$\begin{array}{c} F_{i}^{\prime }=F_{i}\left(1-\eta \alpha_{i}\cdot \frac{F_{i}}{{\left|F_{i}\right|}^{2}}\right), \end{array}$$

where *η* is the feedback coefficient, representing the scale of the force's reduction induced by the displacement *α*_*i*_. *F*_*i*_ is the external force in the previous iteration while *F*_*i*_′ is the newly updated force. With this force correction strategy, the collision detection is needed to be performed only once, which means a great relief of computational burden. With proper setting of the feedback coefficient, not only could the external force be corrected efficiently but also the joint points could approach their equilibrium positions in less iteration.

### 2.5. Graphic Rendering

In our virtual simulation environment, the graphic rendering is heavily relying on modern GPU, which makes the interactive simulation achieve a real-time visualization. In modern GPU, data could be accessed in any stage of the latest programmable pipeline yet it is hardly possible in old, deprecated fixed rendering pipeline. This provides great flexibility to process the geometry data on GPU, taking full advantage of its high performance computing.

To simulate the X-ray imaging, we calculate the linear thickness of the vascular model by fetching the transformed geometry data in the perfragment processing stage with the help of floating-point texture attached multiple render target technique. Based on the following equation which approximates the attenuation of the X-ray imaging, we could get the grayscale of every final pixel on the screen:

(8) $$\begin{array}{c} I=e^{-\sigma *u}, \end{array}$$

where *I* is the grayscale of the target pixel. *σ* is the attenuation coefficient of different objects. *u* is the thickness of the object in the corresponding pixel.

Utilizing the programmability of modern GPU, the Blinn-Phong shading model is implemented in the fragment shader to achieve more realistic and fine visual effect. It means that lighting calculation is postponed to the perfragment processing stage instead of being done in the pervertex processing stage as in the old fixed pipeline. Besides, some other advanced rendering techniques are also employed in our rendering engine to enhance the realism of the simulation environment and to accelerate the interactive rendering speed. For example, geometry data of the anatomical models is transferred to GPU memory, stencil buffer-based shadow volume algorithm is applied to generate high-quality shadows of certain models, and screen space ambient occlusion technique is explored to achieve more realistic endoscopic effect.

### 2.6. Hardware Component

The interface of our hardware component is specially designed in such a way that trainees could use real guide wires and catheters to simulate the requirements of the PCI procedure. We believe that such a user-friendly design will shorten the time to get familiar with instrumental manipulation. The hardware component involves a haptic controller as well as a motion tracker. The tracker is responsible for measuring the translation (pushing and pulling) and rotation (twisting) of both catheter and guide wire and thus comprises two modules: rotation detection and translation detection. The rotation module includes encoder raster, encoder disc, and dummy revolving hollow rod which is connected to the encoder disc. When manipulating the hollow rod, the rotation will be detected by the encoder grating, and the signal is simultaneously transmitted to the controller to control instrument model rotating in the vascular model. For the translation module, it includes encoder, passive wheel, pressing wheel, and pressure adjusting hand wheel. The translation measurements are synchronously sent to the instrument model in the simulation to compute updated physical behaviors. Mechanically, the guidewire is sandwiched between the pressing wheel and passive wheel connected by the encoder. The pressure adjusting hand wheel is connected to the pressing wheel to adjust clamp force and to adapt to different diameters of the guidewire.

## 3. Results and Discussion

**Figure 5:**
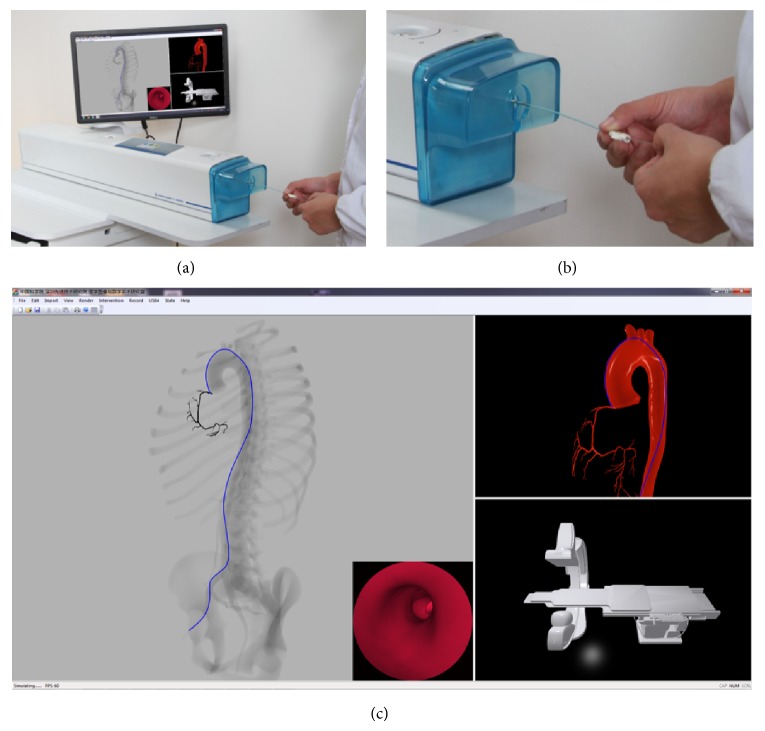
Our simulation system. (a) is the overall view of the system and (b) is a partial interface of the hardware component. (c) is the graphical interface of the software component.

The virtual simulation system we have developed is shown in Figure 5, which is equipped with Windows 7 operating system, running at AMD Athlon II X2 processor at 3.00 GHz with a 4 GB system memory and a NVIDIA Quadro K2000 graphic card with 2 GB video memory. There are four views in the system: fluoroscopic view, visible light view, C-arm view, and endoscopic view. The fluoroscopic view is the major part of the GUI and is responsible for simulating the X-ray imaging. The visible light view is to render the local zoomed region of instruments in a visible light way and consequently its content changes with the position of the instrument's tip, facilitating the display of the motion of the instrument's tip. The C-arm view allows trainees to perform the C-arm with operations such as rotating and translating similar to those in the real catheterization laboratory, and its corresponding effect will be reflected in the fluoroscopic view. The endoscopic view is mounted over the fluoroscopic view in the bottom right region, producing interior views of vasculatures to simulate endoscopic navigation. Although endoscopic camera is not a part of the actual scenario available with the real C-arm device, it can help to choose the desired branch to move on in an intuitive manner when instruments are advanced to bifurcations.

*Hand-Eye Coordination of Instrumental Manipulation*. The simulation environment allows the trainee to manipulate the real catheter and guide wire with one or both hands. As encountered in clinical PCI procedure, two instruments could be maneuvered at the same time in our simulator, with a guide wire inside a catheter, as illustrated in Figure 5(b). The trainee can push the instrument for forwarding, pull the instrument for withdrawing, and twist for rotating. From an entry point in the femoral artery, the instrument (e.g., in blue in Figure 5(c)) could be navigated to the target location of the orifice of the coronary artery by a combined sequence of the aforementioned operations. At this position, the trainee can apply contrast injection to confirm the instrument's position and then make decisions for further steering the instruments to the targeted diseased lesions at coronary artery. In our simulation environment, information about the instrument's tip and the geometry of the local vessels could be obtained with the aid of the visible view and the endoscopic view.

In addition, our simulator is capable of accepting typical types of guidewire with diameters of 0.254 mm to 0.965 mm and catheter with diameters of 1.33 mm to 2 mm, both with lengths ranging from 60 cm to 260 cm. Therefore, trainees could acquire their knowledge and experience on how to select types of both guide wire and catheter at certain PCI phases or according to the situations of vascular lesion.

When contrast dye is injected, the fluoroscopic view can demonstrate vessel (as shown in Figure 5), assisting interventional radiologists in decision making and confirmations. However, as previously mentioned, in clinical procedure, contrast dye injection is performed only when really necessary due to the radiation overexposure to both surgeons and patients. Thus, to prevent trainees from abusing the contrast dye and developing a bad habit of too much relying on fluoroscopic images, the volume and times of contrast used are predefined in the system. When being over the defined threshold, a warning will be popped to remind the trainee that contrast cannot be used any more, although in our system the simulated fluoroscopic imaging is radiation-free and the dye-contrast is poison-free.

When manipulating the real instrument in our virtual environment, trainees should be careful and not exert too much force on one end of the instrument to translate the instrument, because further movement of the instrument may penetrate the vessel walls. In real PCI procedures, such penetration is an intraoperative event and may cause serious damage to the patient. Therefore, a boundary force is predefined in the proposed physical model of instruments to approximate the rupture strength of the vessel wall. If the elastic force applied on the instrument's tip by the vessel wall is greater than the boundary force, the penetration occurs. Therefore, in our system, when the limit of the boundary force is exceeded, the penetration will take place. Such simulation can help trainees see potential disastrous consequences arising from medical mistakes and enable them to experience such events and learn how to manage intraoperative events, which is also a very important skill required in the real catheterization laboratory.

**Figure 6:**
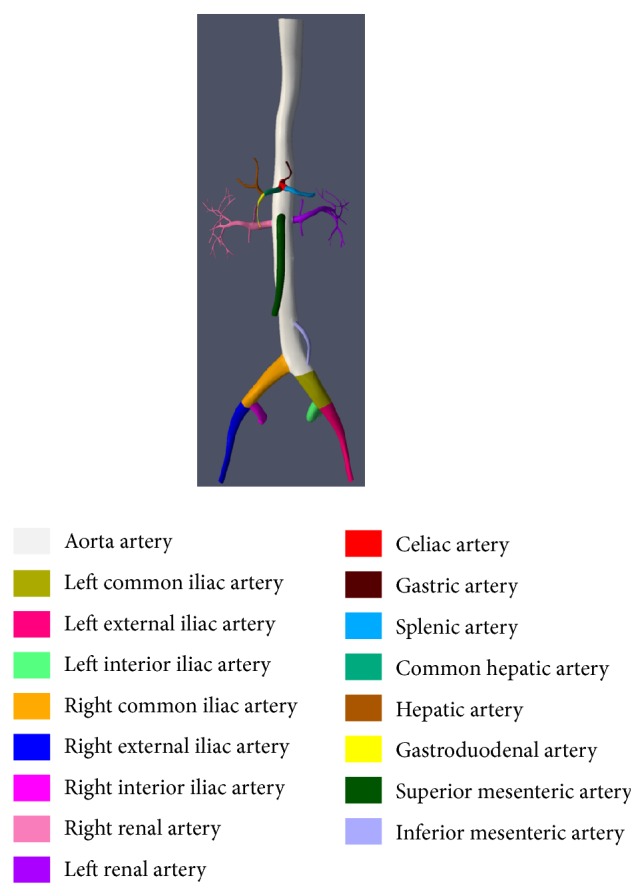
Interface of vascular anatomy learning module.

*Learning Vascular Anatomy*. Like ICTS [4], the presented simulator offers a module of learning. However, in the current development, the learning module only includes vascular anatomy. Based on the extracted centerline of a vascular structure, the vessel surface can be decomposed into several meaningful parts. By hierarchy labeling, the trainee could learn the vascular anatomy in a game of jigsaw puzzle, as illustrated in Figure 6.

**Figure 7:**
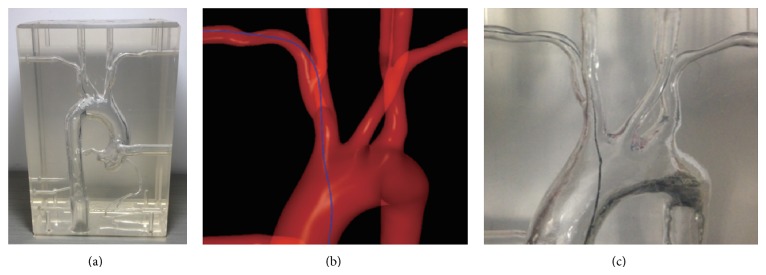
Comparisons of guide wire behaviors on real phantom and virtual model. From (a) to (c) are phantom model, virtual guide wire in virtual model, and real guide wire in real model.

*Initial Validation*. To evaluate the simulation accuracy of the proposed physical model of instruments, validation experiments are carried out on an aorta-coronary phantom (Figure 7) which is made of transparent silicon tubing. Therefore, real instruments could be inserted into the vasculature. The phantom is derived from real human anatomy and thus provides a realistic environment for evaluating our simulated endovascular procedures. The vessel wall of the phantom is rigid, to be the same as our virtual physical model supposes. This hypothesis is reasonable as the instrument's tip is generally made of a kind of very soft flexible material compared to the vessel wall. In our experiments, the phantom is first scanned by computed tomography with a spatial resolution of 0.75 mm and then a semiautomatic segmentation is applied on the 2D scanned images, followed by manual refinement by an expert physician. Finally, 3D vascular surface geometry is obtained by the technique described in Section 2.2.

We perform the same operations that navigate the instrument (guide wire in this experiment) to the same position on the real phantom as well as the reconstructed model. The same operations are preformed several times to guarantee that the guidewire configuration could be reproduced in the physical experimental setup. Meanwhile, corresponding pictures on different stages are taken to make comparisons. Figure 7 shows the comparisons of the guidewire behaviors in the real phantom and the virtual model. Due to the refraction of the transparent silicon material, it is impossible to measure the actual distances of the guidewire between the vessel walls. However, the relative distance, the shape of the guidewire, and the main collision points in pictures indicate that the behaviors of the virtual instruments in virtual models and the behaviors of the real ones in the real vascular phantoms are well matched visually.

*Time Performance*. Timing performance experiments with guide wire represented by different numbers of nodes are carried out (Table 1). It is observed that with moderate discretization of virtual instruments the presented system can achieve real-time interactive visualization and high frame per second (FPS). For a scene with large amount of triangles, for example, the scene shown in Figure 5 containing 623805 triangles (including vascular models, skeleton model, and C-arm model), our system still maintains nearly 60 FPS, which is suitable for an interactive simulation environment. The frame rate includes X-ray rendering, collision detection and response, endoscopic camera, guide wire/catheter deformation, and its visible light rendering.

## 4. Conclusion and Perspectives

In this paper, a realistic and real-time interactive computerized simulation system for training core skills of PCI is presented, accompanied by giving a series of new approaches for high-quality anatomical representations, curve skeleton extraction, and physically based instrument modeling. Various rendering techniques are applied to ensure a real-time visualization by taking full advantage of the GPU computing capability. The characteristics of this system are high-fidelity visual feedback and fast and robust simulation of the instrument behaviors in vasculatures with complex topology and various sizes. The trainee can interact with the virtual simulator with real instruments and essential maneuvers encountered in real catheterization procedures, such as pulling, pushing, and twisting. Most other PCI procedures including C-arm handling as well as contrast dye injection could also be performed in the simulation environment. Therefore, the trainee can acquire the necessary skills such as hand-eye coordination of catheter/guide wire navigation and fluoroscopic imaging. Although presently targeted at PCI procedure, our system has the potential to mimic principle necessities of any interventional radiology procedures by simply updating vascular anatomy.

We are in a process of modeling vascular hemodynamics with the pathology model of cardiac vasculature [27], which could be used to simulate blood flow phenomenon in the areas of vessel stenoses and malformations. At present, the hemodynamic model is independent but will be seamlessly incorporated into the system soon. A limitation of the current physical model of instruments is the lack of taking into account the interactions between blood flow and instruments. Once the blood flow simulation is added, the physical model will then be enhanced.

In addition, a set of performance metrics established by highly experienced interventional cardiologists will be integrated into the system to record the correct and incorrect actions of the trainee. Furthermore, when a trainee makes an error, the consequences will be reflected in the system. For example, if the vessel wall is penetrated by the instruments, then hemorrhage will occur and be simulated. By this way the trainee can see potential consequences on virtual patient and learn how to avoid and manage intraoperative catastrophic events. In clinical procedures, it is impossible to ensure that trainees experience full range of intraoperative complication management because complications and adverse operative events are not common. Therefore, complication simulation is especially crucial to a valuable virtual environment and will need more efforts in our future works, aiming to offer opportunities for trainees to react to rare complications.

The current vascular patient database will be added to as many as possible diseased cases as different cases (e.g., different diseased sites or different levels of diseased lesion) need different surgical strategies, different manipulation techniques, and different types of instruments. Rich diversity in cases will not lead to boredom for trainees. Furthermore, a vascular editing tool will be developed, with which trainees can change the geometry and characteristic of the vasculature such as changing the diameter of the vessel to mimic a blockage or narrowing or changing the diseased location. We believe that such tool will greatly help to cover enough diseased cases and help trainees to get familiar with the requirements of various cases. The system will be beneficial to senior cardiologists when developing new intravascular devices as well as surgical procedures.

Currently, our prototype has been validated in terms of the physical modeling of instruments. However, upon the completion of several additional components that are ongoing development, it will undergo face and content validity from our hospital collaborators and then a comprehensive assessment and validation study including concurrent and predictive validity.

As the ultimate goal of the presented system is not limited to providing interventional radiologists training and learning environment with high realism, high immersion, and user friendliness but also offering an accurate pretreatment planning tool for interventional radiologists in preparation for catheterization procedures with patient-specific image data, we will explore the integration of an automatic vascular segmentation or a semiautomatic approach with minimum extra tasks into the current prototype.

## Figures and Tables

**Table 1:** Time performance for our system.

Number of nodes	Collision response (ms)	Frame per second
40	1.1	210
60	3.4	195
80	5.2	179
100	8.9	155
120	17.9	110
140	23.3	57
